# Editorial Note: Soil quality indicator-based land productivity modelling for agricultural sustainability

**DOI:** 10.1371/journal.pone.0351483

**Published:** 2026-06-12

**Authors:** The PLOS One Editors

The *PLOS One* Editors issue this Editorial Note for this article [1] because of concerns identified about the peer review process. Readers are advised to interpret the article [1] with caution.

The Editors have no evidence of any author involvement in the peer review concerns.
